# Somatic mutations of esophageal adenocarcinoma: a comparison between Black and White patients

**DOI:** 10.1038/s41598-024-59257-3

**Published:** 2024-04-18

**Authors:** Hyeyeun Lim, Marie-Claude Gingras, Jing Zhao, Jinyoung Byun, Patricia D. Castro, Spiridon Tsavachidis, Jianhong Hu, Harshavardhan Doddapaneni, Yi Han, Donna M. Muzny, Richard A. Gibbs, Christopher I. Amos, Aaron P. Thrift

**Affiliations:** 1 Section of Epidemiology and Population Science, Department of Medicine, Baylor College of Medicine, Houston, TX USA; 2 Human Genome Sequencing Center, Department of Molecular and Human Genetics, Baylor College of Medicine, Houston, TX USA; 3 Department of Pathology, Baylor College of Medicine, Houston, TX USA; 4 Dan L Duncan Comprehensive Cancer Center, Baylor College of Medicine, Houston, TX USA; 5 Institute for Clinical and Translational Research, Baylor College of Medicine, Houston, TX USA; 6 Dan L Duncan Comprehensive Cancer Center, Baylor College of Medicine, One Baylor Plaza, MS: BCM307, Room 621D, Houston, TX 77030 USA; 7 Institute for Clinical and Translational Research, Baylor College of Medicine, One Baylor Plaza, MS: BCM451, Suite 100D, Houston, TX 77030 USA

**Keywords:** Cancer, Genetics, Gastroenterology

## Abstract

Esophageal adenocarcinoma is the most common histological subtype of esophageal cancer in Western countries and shows poor prognosis with rapid growth. EAC is characterized by a strong male predominance and racial disparity. EAC is up to fivefold more common among Whites than Blacks, yet Black patients with EAC have poorer survival rates. The racial disparity remains largely unknown, and there is limited knowledge of mutations in EAC regarding racial disparities. We used whole-exome sequencing to show somatic mutation profiles derived from tumor samples from 18 EAC male patients. We identified three molecular subgroups based on the pre-defined esophageal cancer-specific mutational signatures. Group 1 is associated with age and *NTHL1* deficiency-related signatures. Group 2 occurs primarily in Black patients and is associated with signatures related to DNA damage from oxidative stress and *NTHL1* deficiency-related signatures. Group 3 is associated with defective homologous recombination-based DNA often caused by *BRCA* mutation in White patients. We observed significantly mutated race related genes (*LCE2B* in Black*, SDR39U1* in White) were (q-value < 0.1). Our findings underscore the possibility of distinct molecular mutation patterns in EAC among different races. Further studies are needed to validate our findings, which could contribute to precision medicine in EAC.

## Introduction

Esophageal cancer is one of the common cancers and was responsible for 5.5% of all deaths from cancer worldwide in 2020^1^. There are two main histological subtypes of esophageal cancer, esophageal adenocarcinoma (EAC) and esophageal squamous cell carcinoma (ESCC). In Western countries, EAC is the predominant subtype, representing 40–70% of all esophageal cancers^2,3^. In the United States, EAC incidence has increased substantially over the last four decades, and absolute rates of EAC have remained highest among non-Hispanic White males^3^. While there have been improvements in therapies and surgery, fewer than 20% of EAC patients survive for five years post-diagnosis^4,5^. Although Black patients have a lower incidence, the average onset age of advanced cases is younger, and survival rates are lower than White patients^6,7^. Gastroesophageal reflux disease (GRED) symptoms, smoking, obesity, and low socio-economic status are well-known risk factors of EAC but are unlikely to explain fully the differences in incidence and outcomes between Black and White patients^2,4,5,8^.

Previous studies have revealed that EAC is a heterogeneous cancer with a high mutational burden in *TP53, CDKN2A, SMAD4, APC, KRAS, MYC, ERBB2, GATA4, PTEN* and *ARID1A*, and with high chromosomal instability^9–12^. Somatic mutations of *TP53, SMAD4*, and *GATA4* have been reported to be associated with poor prognosis in EAC^13–15^. A dominant A > C at AA dinucleotides mutational pattern has been found in EAC and is associated with acid reflux^10,12,16^. However, these studies have been conducted among exclusively White EAC patients.

According to a study of ESCC using The Cancer Genome Atlas Research Network study, ESCC has clustered into three main molecular subgroups based on somatic mutation profiles^17^. Group 1 is characterized by oxidative stress, the amplification of transcription factors *TP63* and *SOX2*, and has been found primarily in Asian patients. Group 2 has shown higher rates of mutations of *NOTCH1, ZNF750, KDM6A*, and *KMT2D* and occurs primarily in European patients. Group 3 is found in a few patients (N = 4) that displayed high somatic alterations in *PTEN, PIK3A,* and *SMARCA4* but infrequently have *TP53* mutations.

A study of Chinese EAC patients has shown a significantly lower mutation burden and median percentage of hallmark A > C mutational signature than those in US/UK EAC^18^. However, knowledge of mutations in EAC regarding racial disparities is limited. Hence, the differences in survival outcomes and tumor biology within the EAC between Black and White patients remain to be fully understood.

In our current study, we sought to assess the somatic mutation profiles derived from formalin-fixed paraffin-embedded (FFPE) tumor samples from male patients with EAC by conducting whole-exome sequencing (WES). We performed somatic mutational signature analysis to compare the pattern of single nucleotide variants (SNVs) in EAC samples to pre-defined esophageal cancer (ESCA)-specific signatures from the SIGNAL database^19^. We identified signatures that differentially contributed to EAC mutagenic processes by race with the goal of obtaining the genomic landscape of EAC and its underlying mutational processes. In addition, we identified differentially mutated genes and compared the mutation load between Black and White patients.

## Results

### Patient and tumor characteristics

Twenty-one EAC samples were sequenced. The characteristics of the patient and tumor are shown in Supplementary Table 1. The mean age is 69.4 years, ranging from 52 to 90 years. Eleven are Black patients. Fourteen (66%) tumors were graded as poorly differentiated or moderately to poorly differentiated.

### Somatic mutation profiling in EAC

**Figure 1:**
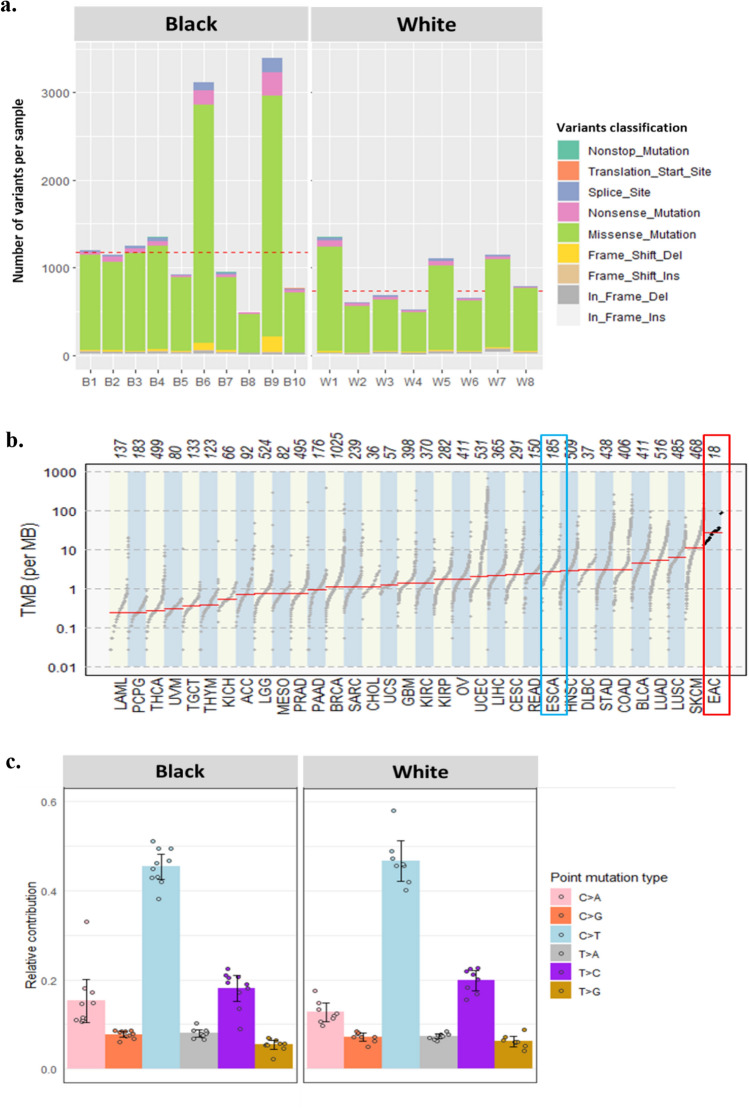
Summary of exome sequencing and TMB. (**a**) Number and types of variants found in each sample. The red dashed line presents the median of the total number of variants (Black = 1172, White = 735.5) (**b**) The comparison of mutation burden of 18 EAC samples across TCGA database. Our samples are indicated by the red rectangle. Esophageal cancer samples in TCGA database are indicated by the blue rectangle. (**c**) Proportion of somatic substitution types in processed and filtered SNVs/Indels. Dots represent individual samples.

Of the 21 samples, three showed read depth < 50× and were excluded from further analysis. We identified a median of 1024 mutations per sample (range: 492–3395), corresponding to a median tumor mutation burden (TMB) of 27.6/MB (range:13.3/MB – 91.8/MB) (Fig. 1a, Supplementary Table 1). By race, the median variant of Black patients was 1.6-fold higher (N = 1172) compared to White patients (N = 735.5) but was not statistically significant (Wilcoxon Rank Sum test *p*-value = 0.11). Two samples displaying a high TMB/MB were from Black patients (B9 and B6). In these samples, the average number of variants was 3256.5. In contrast, low TMB was detected in two and one samples from White (W2 and W4) and Black patients (B8), respectively (average number of variants = 1617) (Fig. 1a).

Missense mutations (N = 18,693) constituted the primary type of alteration in the coding region of tumor samples. Nonstop (stop-loss) was rare (N = 23) (Fig. 1a, Supplementary Table 2). The observed median TMB/MB (median = 27.6) was higher than that observed in UK EAC patients (median = 20.6/MB) and 3 to 10 folds higher than that previously reported in the US (median = 9.9/MB) and Chinese (median = 2.56/MB) EAC patients, respectively^12,18^. In our study, the median TMB/MB in Black and White patients was 31.7 and 19.8, respectively. The median TMB/MB in White patients was very similar to the median TMB/MB of the UK EAC study (median = 20.6/MB), which mainly consisted of White patients. Compared to the TMB/MB of 30 different cancers in the Cancer Genome Atlas Program (TCGA) database, the TMB/MB of this study was the highest among the 30 different cancers (Fig. 1b). A study of African American (AA) ESCC study also observed the median mutation rate was greater the most tumors in TCGA and located between skin cutaneous melanoma and lung squamous cell carcinoma^20^.

When we classified the distribution of SNVs into six transitions, C > T transversion was predominant in most samples (Fig. 1c). This finding is consistent with previous studies of EAC patients in the US and China^18,21^. Overall, T > G transversion accounts for 6% of total mutations, similar to the proportion in Chinese EAC but threefold lower than in the US EAC study^18^. C > G transversion only showed a significant difference between Black and White EAC patients (Wilcoxon Rank Sum test *p*-value = 0.01). Smoking cigarettes or the patient’s age causes oxidative stress and inflammation. However, in our study, the smoking status (never, former, current smoker) and the age at diagnosis did not significantly differ by race and point mutation type (correlation coefficient = 0.01, *p*-values using the Wilcoxon Rank Sum test for smoking status at diagnosis > 0.05).

### Mutational signatures in EAC

**Figure 2:**
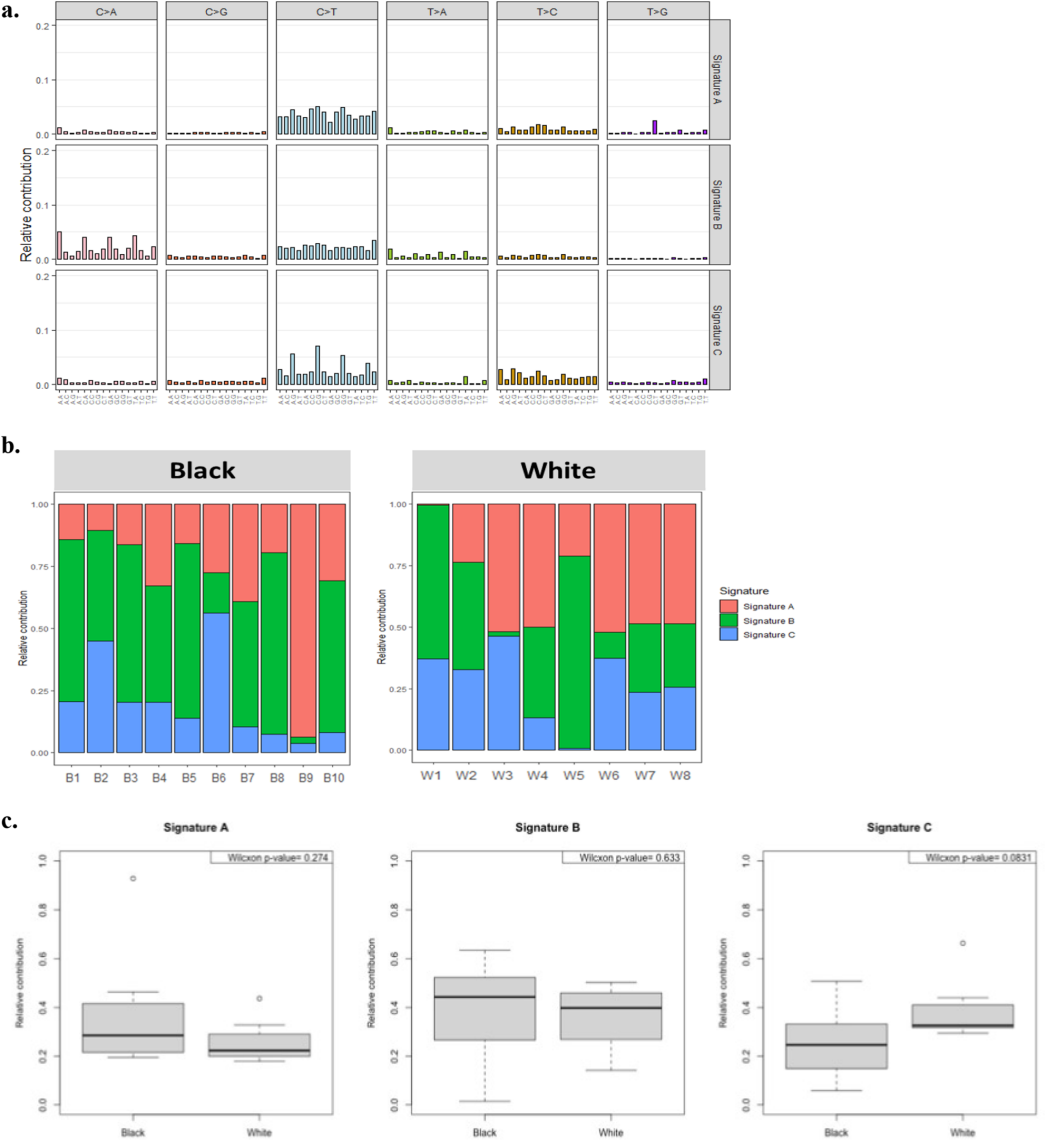
Mutational Signatures were identified using a NMF algorithm. (**a**) The relative contribution of each indicated trinucleotide change to the three mutational signatures that were identified by NMF analysis of the somatic mutation of the EAC samples (**b**) Bar graphs show the percentage of the three signatures in the EAC tumor samples. Signature A was found in a very high proportion in sample B9, but Signature B and C were found in a very low proportion. Signatures A and C were found in very low proportion in samples W1 and W5, respectively. (**c**) Comparison of the relative contributions with race. Wilcoxon rank‐sum test was used.

**Figure 3:**
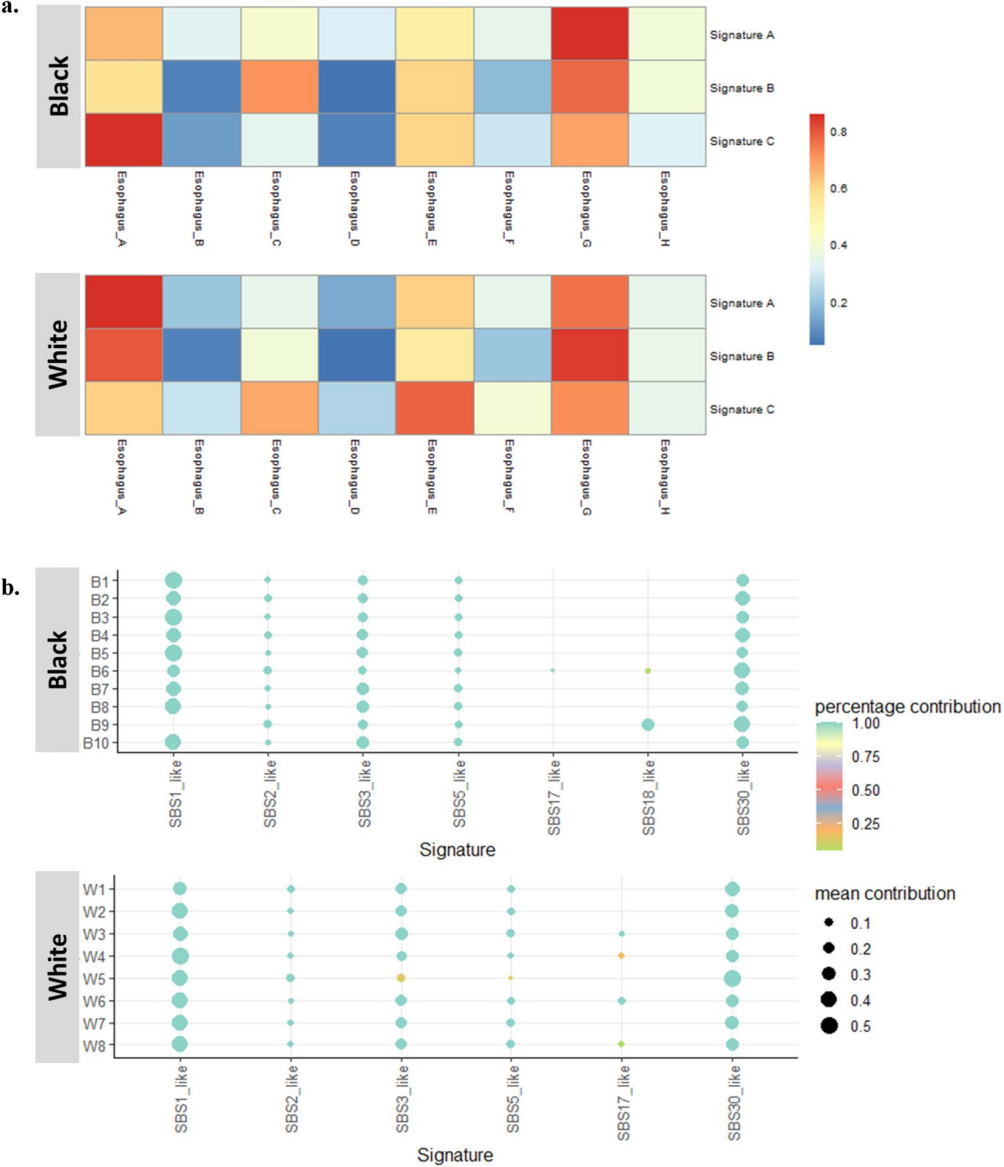
Mutational signatures based on ESCA-specific mutational signatures from SIGNAL. (**a**) Heatmap shows cosine correlations between the three mutational signatures of this study cohort and ESCA-specific mutational signatures from the SIGNAL database. The correlation coefficient r = 1 is red, r = 0 blue color. (**b**) Dotplot of the relative contribution of individual EAC cases compared with ESCA-specific signatures from the SIGNAL database. The color of the dot shows the percentage of iterations in which the signature is found (contribution > 0), and the size of the dot represents the average contribution of that signature (in the iterations in which the contribution was higher than 0).

We performed somatic mutational signature analyses with a set of SNVs using a non-negative matrix factorization (NMF) algorithm and extracted three mutational signature subgroups. MutationalPatterns package was used to quantify the contribution of mutational signatures. C > T substitution was predominant in the three signature groups (Fig. 2a). Signature B demonstrated significant C > A transversion compared to Signature A and C. Signature C demonstrated significant T > C transversion compared to Signature A and B. Figure 2b shows the percentage of the three signature groups per individual sample by race. A sample from Black patients (B9) showed a very high proportion of Signature A, but Signature B and C were found in a very low proportion. Among the samples from White patients, Signature A and C were found in high proportion in most of the samples but very low proportion in two samples (W1 and W5). Two samples from Black patients (B2 and B6) and one sample (W3) from White patients displayed the highest contribution to Signature C (relative contribution > 0.4). Comparison of the relative contributions of each mutational signature with race showed no statistically significant differences between Signatures and race in EAC samples (Wilcoxon rank‐sum test *p*-value > 0.05) (Fig. 2c). We calculated the similarity between the three observed mutational signatures (Signature A, B, and C) and eight pre-defined esophageal cancer-specific mutational signatures from the SIGNAL database (https://signal.mutationalsignatures.com) (Fig. 3a, Supplementary Table 3)^22^. Among Black patients, Signature A showed a strong correlation with ESCA G signature (cosine-correlation = 0.89). Signature B was correlated with ESCA C and G signatures (cosine-correlation = 0.73 and 0.80, respectively). Signature C was found to be related to ESCA A and G signatures (cosine-correlation = 0.88 and 0.70, respectively). Similarly, White patients were grouped into ESCA A and G related group and ESCA E and G related group. We compared the ESCA-specific signatures with COSMIC 96 signatures to define the etiology and then named the most similar COSMIC signatures (Fig. 3b). ESCA A, B, C, D, E, F, G, and H groups were the most similar to COSMIC signatures SBS1, SBS17b, SBS18, SBS17b, SBS3, SBS28, SBS30 and SBS2, respectively (Supplementary Table 4). We quantified the contribution of the eight ESCA-specific signatures to the mutational profile of individual EAC samples in this cohort (Fig. 3b). The signature correlating mostly with the age of cancer diagnosis (SBS1-like) and NTHL1 deficiency-related signature (SBS30-like) predominately contributed to the mutation spectrum of EAC tumors regardless of race. SBS18-like was reported to be associated with DNA damage caused by reactive oxygen species. In our study, only Black patients with high TMBs (B6 and B9) showed a correlation. The SBS3-like signature was the most correlated with Signature C in White patients (cosine correlation = 0.78). The defect of homologous recombination (HR)-based DNA caused by BRCA1 and/or BRCA2 mutation or pathway-level disruption of HR contributes to the SBS-like signature ^10,23^. The UK EAC study observed three signature groups of patients: SBS18-like (age), SBS3-like (*BRCA*), and predominantly SBS17A or SBS17B (mutagenic), which are similar in composition to those seen in our study^10^.

We calculated the similarity between the three observed mutational signatures (Signature A, B, and C) and 96 COSMIC signatures (Supplementary Fig. 1). COSMIC Signature (SBS) 1, SBS5, SBS6, SBS7b, SBS87, and SBS89 were observed across most of the EAC samples. To compare the differences by race, we performed the Wilcoxon rank test. Although we observed *p*-value < 0.05 for SBS26 (Wilcoxon rank‐sum test *p*-value = 0.04) and SBS33 (Wilcoxon rank‐sum test *p*-value = 0.03), these were not statistically significant after Bonferroni adjusted significant tests (Bonferroni adjusted *p*-value = 0.05/30).

### EAC cancer-related genes

**Figure 4:**
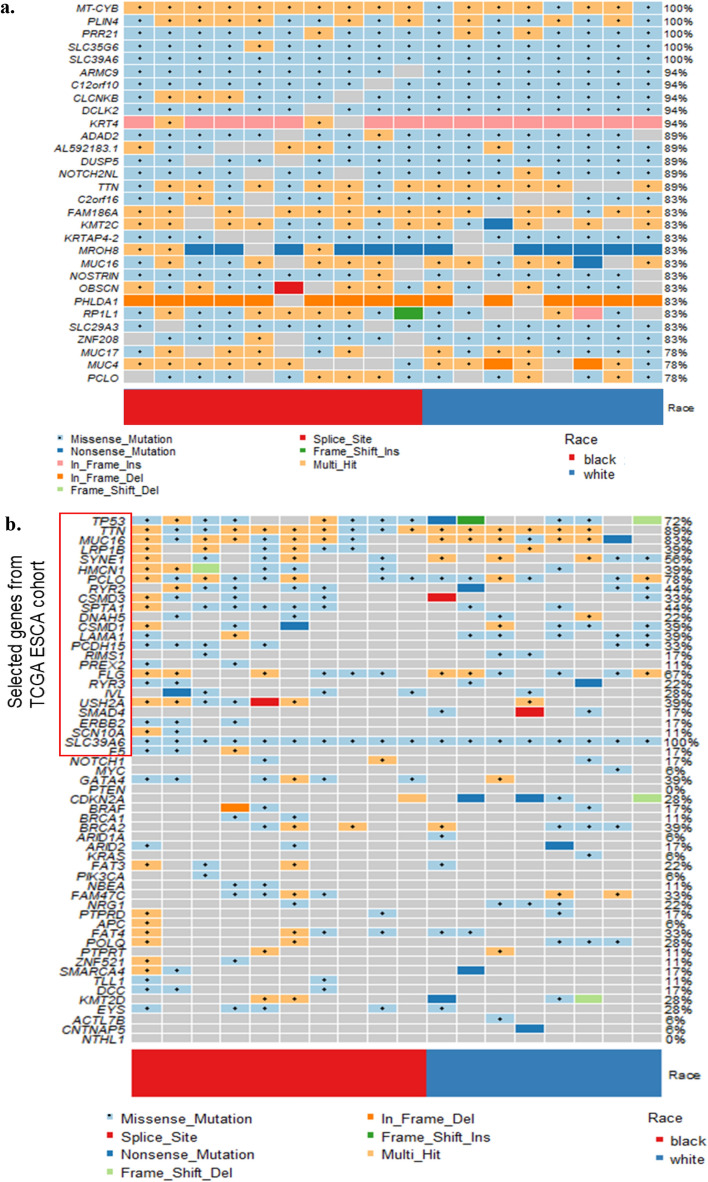

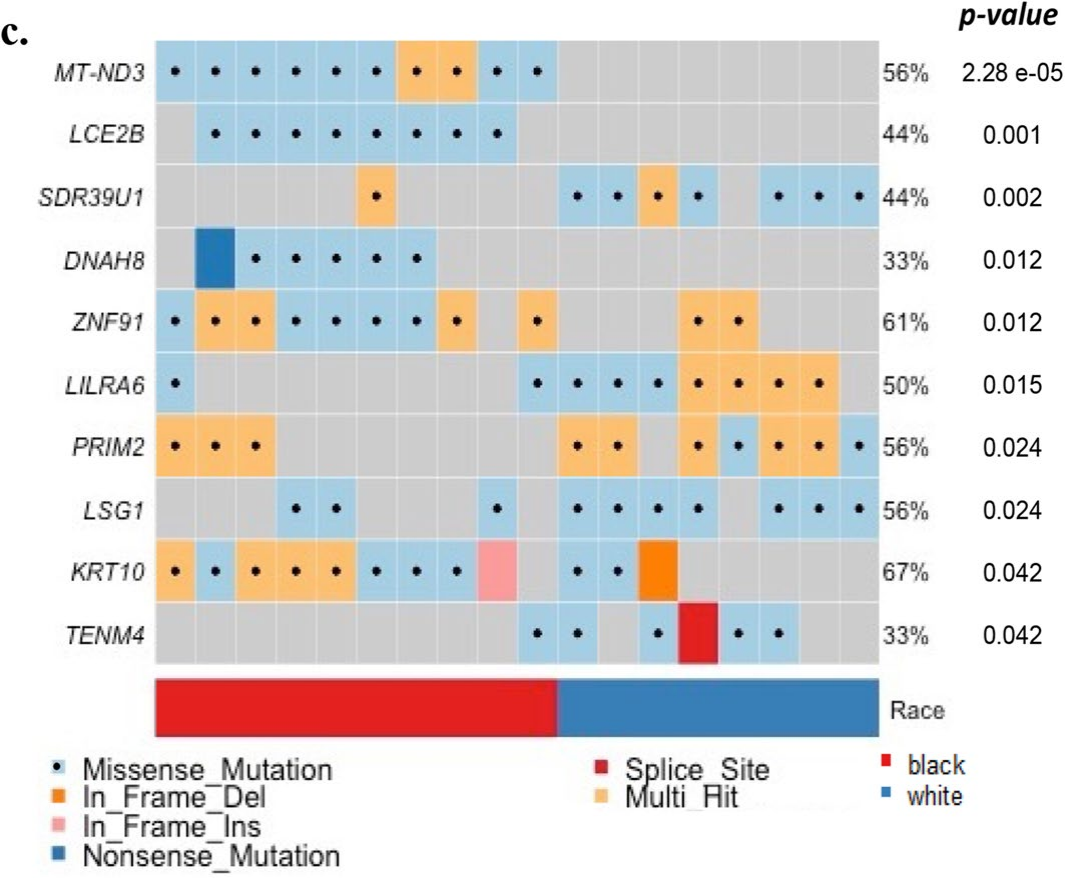
Single nucleotide variants and InDels of EAC. Oncoplot by maftools visualized mutations of missense, frameshift InDels, nonsense, and splice site. (**a**) The top 30 frequently mutated genes were plotted. Genes are ordered by their mutation frequency, and samples are ordered according to race as indicated by the annotation bar (bottom). (**b**) Oncoplot of EAC driver genes from TCGA ESCA cohort and literature. The genes from TCGA ESCA cohort are indicated by the red rectangle. (**c**) The ten genes according to the *p*-value < 0.05 from Fisher’s exact test for comparing the number of patients affected by individual genes between Black and White patients.

We observed mutations in 9,662 genes, of which 4,769 were mutated in two or more patients (Supplementary Table 2). Figure 4a shows the top 30 frequently mutated genes by SNVs/Insertions and deletions (InDels) type and race. *MT-CYB* (Mitochondrially Encoded Cytochrome B), *PLIN4* (Perilipin 4), *PRR21* (Proline-rich protein 21) *SLC35G6* (Solute Carrier Family 35 Member G6) and *SLC39A6* (Solute Carrier Family 39 Member A6) were found in all patients. *MUC4, MUC16,* and *MUC17* were found in most tumor samples ( > 78%). Those Mucin genes are known to have anti-oxidation roles and are associated with poor cancer survival in pancreatic, colon, and stomach cancer^24,25^. Mutations in *KRT12* consist of dominant in-frame insertion in tumor samples from White patients. In-frame deletion in *PHLDA1* gene was found in most samples (15/18 samples). *ERBB2, F5, PREX2, BRCA1*, and *SCN10A* were observed in only Black patients. *SMAD4* was mutated in only White patients (3/8 samples). In addition, we selected the most mutated genes in EAC patients from the TCGA ESCA cohort using the National Cancer Institute GDC Data portal and genes previously reported in the literature (Table 1 and Fig. 4b)^10–12,16,26–30^. Of the 50 selected genes, *TP53, TTN, MUC16, PCLO*, and *SLC39A6* were mutated in > 70% of patients in our study. The frequency of *TP53* mutation of White patients in our study was slightly lower than that of White EAC male patients in the TCGA cohort. This finding is consistent with a British whole-genome study (WGS) of 129 EAC patients^10^. The average mutation frequency of *TP53, TTN, MUC16, PCLO*, and *SLC39A6* was twice as high among White patients compared to the TCGA cohort. We compared the results of our study on White male EAC patients with those in the TCGA ESCA cohort, as the TCGA cohort did not have any data on Black EAC patients. To eliminate false-positive findings from a small sample size and high mutation rate, we used MutSigCV 2.0 to identify significantly related genes^31^. This tool estimated the background mutation rate (BMR) for each gene-patient-category by comparing the silent mutation occurrence in each gene to the non-coding mutations in the surrounding genes. There were 68 genes that had *q*-value < 0.1 after correction muti-comparison and a mutation frequency greater than 20%. *TP53*, *CSMD3*, and *CDKN2A* were identified as significantly mutated genes. Otherwise, *TTN*, *MUC16*, and *PCLO* were not. All the genes are shown in Supplementary Table 6. In addition, we compared the number of Black patients affected by individual genes with those of White patients using a two-tailed Fisher’s exact test (Fig. 4c). *LCE2B* and *SDR39U1* were significantly mutated genes after correction for multiple comparisons (*q*-value < 0.1).

**Table 1:** Number of esophageal adenocarcinoma (EAC) patients by race and mutated genes in our EAC study and TCGA esophageal cancer (ESCA) cohort.

Gene	TCGA ESCA cohort^a^	EAC study	
	White male (N = 60) (%)	Black male (N = 10) (%)	White male (N = 8) (%)
TP53	48 (80.0)	8 (80.0)	5 (62.5)
TTN	35 (58.3)	10 (100.0)	6 (75.0)
MUC16	21 (35.0)	8 (80.0)	7 (87.5)
LRP1B	21 (35.0)	6 (60.0)	1 (12.5)
SYNE1	17 (28.3)	5 (50.0)	5 (62.5)
HMCN1	15 (25.0)	6 (60.0)	1 (12.5)
PCLO	14 (23.3)	8 (80.0)	6 (75.0)
RYR2	14 (23.3)	5 (50.0)	3 (37.5)
CSMD3	13 (21.7)	4 (40.0)	2 (25.0)
SPTA1	13 (21.7)	6 (60.0)	2 (25.0)
DNAH5	13 (21.7)	2 (20.0)	2 (25.0)
CSMD1	12 (20.0)	3 (30.0)	4 (50.0)
LAMA1	11 (18.3)	2 (20.0)	5 (62.5)
PCDH15	11 (18.3)	4 (40.0)	2 (25.0)
RIMS1	11 (18.3)	1 (10.0)	2 (25.0)
PREX2	11 (18.3)	2 (20.0)	0
FLG	10 (16.7)	6 (60.0)	6 (75.0)
RYR3	10 (16.7)	2 (20.0)	2 (25.0)
IVL	10 (16.7)	4 (40.0)	1 (12.5)
USH2A	9 (15.0)	6 (60.0)	1 (12.5)
SMAD4	7 (11.7)	0	3 (37.5)
ERBB2	6 (10.0)	3 (30.0)	0
SCN10A	2 (3.2)	2 (20.0)	0
SLC39A6	1 (1.6)	10 (100.0)	10 (100.0)
F5	0	3 (30.0)	0

^a^There are no Black EAC patients in the TCGA esophageal cancer cohort.

### EAC cancer mutation signatures and related genes after excluding high TMB samples

We sought to assess mutation signature and EAC-related genes after excluding the two outlier samples (B6 and B9) with high TMB/MB. The median mutation per sample was 935 (range: 492–1346), corresponding to a median TMB of 18.7/MB (Supplementary Fig. 2a). By race, the median variant and TMB/MB of Black patients were 1047 and 28.3/MB, respectively. Missense mutations constituted the primary type of alteration in the coding region of tumor samples. Nonstop (stop-loss) was rare. We extracted two mutational signature subgroups using the NMF algorithm. C > T substitution was predominant in the two signature groups (Supplementary Fig. 2b). C > G transversion only showed a significant difference between Black and White EAC (Wilcoxon Rank Sum test *p*-value = 0.03). The percentage of the two signature groups per individual sample by race was shown in Supplementary Fig. 2c. Comparison of the relative contributions of each mutational signature with race showed no statistically significant differences between Signatures and race in EAC samples (Wilcoxon rank‐sum test *p*-value > 0.05, data not shown). Regardless of the patient's race, SBS1-like and SBS30-like signatures were the primary contributors to the mutation spectrum of EAC tumors, followed by SBS3-like (Supplementary Table 5). By comparison between the number of Black patients affected by individual genes and those of White patients using a two-tailed Fisher’s exact test, we identified six genes (*MT-ND3, LCE2B, SDR39U1, KRTAP5-7, ZNF91*, and *LILRA6*) with a *p*-value > 0.05 (Supplementary Fig. 3). As like before exclusion, *LCE2B* and *SDR39U1* were only identified as significantly mutated genes after multiple comparisons (*q*-value < 0.1).

## Discussion

Despite EAC rates remaining high among White males, the relative survival rate is lower in African Americans, and there is a paucity of genomic studies in EAC by race. The current study revealed a genomic landscape and mutated genes that may differently contribute to racial disparities of EAC tumorigenesis and survival in males. We have conducted whole exome sequencing for 10 of EAC tumor tissues in Black patients and 8 in White patients, which to our knowledge, is the first comprehensive analysis of genomic alterations and racial disparity in Blacks versus Whites. We identified three molecular EAC subgroups. Group 1 is the most common in EAC and is associated with age and *NTHL1* deficiency-related signatures. Group 2 occurs primarily in Black patients and is associated with signatures related to DNA damage from oxidative stress and *NTHL1* deficiency-related signatures. Group 3 is associated with defective homologous recombination-based DNA often caused by *BRCA* mutation in White patients. We identified *LCE2B* and *SDR39U1* were significantly mutated genes between Black and White racial groups.

A recent analysis of WGS data from 149 White patients in the US reported that C > T is the dominant mutation pattern, which has been reported with evidence of an aging imprint in EAC, followed by T > G mutation pattern^10,12^. Our data reveal the dominant mutation pattern is C > T (median = 47%), and the T > G mutation pattern is the lowest (median = 5%). When comparing the proportions of six possible base changes by race, C > G mutation pattern showed significant differences by race. It was identified more frequently in Black EAC patients compared to White patients. The significant difference in the C > G mutation pattern between the Black and White groups persisted after excluding high TMB samples (Wilcox Rank Sum test *p*-value = 0.03). G-C $\to$ C-G transversion mutations that occur under oxidative conditions because the guanine base(G) in genomic DNA is highly susceptible to oxidative stress. In our study, the C > G point mutation type was not associated with the smoking status and the age at diagnosis. Our study found a high mutation rate when compared to the TCGA database and other research on EAC. The median TMB/MB in White patients (median = 19.8/MB) in our study was very similar to the median TMB/MB of the UK EAC study (median = 20.6/MB), which mainly consisted of White patients and > 60% T3/T4 patients^10^. An AA ESCC study^20^, where 90% of the samples were from AA patients with advanced stage ESCC, showed the mutation burden was greater than that of different cancers in TCGA and was located between lung squamous cell carcinoma and skin cutaneous melanoma^20^. The high mutation rates or burdens could be due to the fixation and archiving process or storage in FFPE, which could lead to the degradation and fragmentation of DNA. However, all the samples were processed, stored in the same place, and sequenced on the same day. There was no relationship between TMB/MB and the time lapse from resection to sequencing (correlation coefficient = 0.05). Studies compared the whole exome sequencing results of FFPE and fresh frozen samples and found a good somatic SNV calls concordance between FFPE and matched fresh frozen tissue samples even though false positive/negative could be caused at the positions with insufficient sequencing depth^32,33^.

Our study used germline mutation data from Genome Aggregation Database (gnomAD). This also could cause false-positive calls and overestimate the mutation burdens, especially in low-frequency germline variants, not all of which may have been reported to gnomAD^34,35^. To overcome the limitation, we called SNVs cutoff of at least two of three variants with read depth of > 50×), variant allele frequencies (VAF) cutoff of > 5%, and minor allele frequency (MAF) cutoff of < 1% for rare variants. A study of somatic mutation detection rate with matched normal tissue and only tumor tissues showed the sensitivity and specificity with only tumor samples for well-known genes was very similar compared to the rate with matched normal^35^. Thus, we compared the number of Black patients who harbored well-known EAC genes to White patients. Another limitation is the small sample size with limited information on risk factors such as pre-existing medical history and smoking behavior. Although we used the MutSigCV 2.0 to eliminate false-positive findings from the small sample size, we cannot eliminate the possibility our patient cohort is not entirely representative of AA EAC patients. Further studies of longitudinal cohorts of EAC patients are needed to validate our findings of discovered genomic alterations and their relationship with environmental exposures and risk factors.

In summary, our present study is the first comprehensive exome sequencing analysis to compare the difference in mutations between Black and White EAC patients using mutational signature analysis. Our findings underscore the possibility of distinct molecular mutation patterns in EAC among different races. Further comprehensive epidemiologic and epigenetics studies are necessary to validate our findings in larger sample sizes and understand the underlying mutational mechanisms of EAC.

## Methods

### Patients and sample collection

WES of FFPE tumor specimens from 21 EAC (Black patients = 11, White patients = 10) was performed. Tissue blocks were identified from a search of the Human Tissue Acquisition and Pathology (HTAP) core at Baylor College of Medicine. The staging of the patients was done according to the methods described in the American Joint Committee on Cancer Staging Manual. This study was approved by both the Institutional Review Board at Baylor College of Medicine and the Michael E. DeBakey Veterans Affairs Medical Center, and all subjects gave individual informed consent to HTAP. Written informed consent and approval for research were obtained from each patient. All methods were performed in accordance with relevant guidelines and regulations.

Race was categorized by patient self-identification. We pulled tissue blocks for all Black men with EAC and paired them with tissue blocks from White men by matching for year of diagnosis and age at diagnosis.

### DNA isolation

The DNA was isolated using QIAamp DNA FFPE tissue kit and following the manufacturer’s protocol (Qiagen).

### Library preparation

FFPE DNA samples were constructed into Illumina paired-end pre-capture libraries according to the 500 ng or 250 ng (depending on sample quantity availability) of DNA in 50ul volume. gDNA was sheared into fragments of the average size of 250–300 base pairs in a Covaris plate with an E220 system (Covaris, Inc. Woburn, MA) followed by end-repair (NEBNext End Repair Module—E6050L) and A-tailing (NEBNext dA-Tailing Module—E6053L), ligated with Illumina Dual index PE adapter using (Life Tech. ExpressLink Ligase- A13726101), Pre-capture Ligation Mediated-PCR (LM-PCR) was performed for 6–8 cycles using the Library Amplification Readymix containing KAPA HiFi DNA Polymerase (Kapa Biosystems, Inc., Cat # KK2612). Universal P1.1 (5′-AATGATACGGCGACCACCGAGA) and P3 (5′-CAAGCAGAAGACGGCATACGAGA) primers were used to amplify the ligated products. AMPure XP beads (A63882, Beckman Coulter) were used for library purification. Quantification and size distribution of the pre-capture LM-PCR product were determined using the Fragment Analyzer capillary electrophoresis system (Agilent Technologies, Inc).

### Capture enrichment

A total of 21 samples were pooled as 10 plex and 11plex (∼200 ng/sample, 2 μg/pool) and then hybridized in solution to the HGSC VCRome 2.1 design provided by NimbleGen based on the manufacturer’s protocol *NimbleGen SeqCap EZ Exome Library SR User’s Guide *(*Version 2.2*) with minor revisions^36^. The detailed capture enrichment process was described previously^37^.

### Whole-exome sequencing

Sequencing was performed on the NovaSeq 6000 instrument using the S4 reagent kit (300 cycles) to generate 2 × 150 bp paired-end reads. Post-capture library pools were sequenced on NovaSeq S4 flow cell to generate between 3.2 and 180.7 Gb unique sequence data per sample (Table 1).

### Analysis of sequences

The processing and analysis of WES data were following Genome Analysis Toolkit (GATK) Best Practices^38^. Paired-end reads were mapped to the human reference genome (hs37d5) using multi-threaded BWA53, and a BAM file was generated for each tumor sample. To assess the alignment quality, the FastQC package was used (http://www.bioinformatics.babraham.ac.uk/projects/fastqc). Three samples that failed quality control threshold (< 50×) were excluded from the final data analysis (Supplementary Table S1). For somatic analysis, the base recalibrated tumor BAMs were fed to Mutect2 (GATK-v4.1)^39^ to call somatic variants at chromosome positions covered in the target bed. We used a list of germline mutations from gnomAD^40^. Variant filtering was done based on the FilterMutectCalls function of GATK tools^38^, and further filtering was done based on read depth (DP > 4). The filtered somatic mutations were annotated using GATK Funcotator^41^. The resulting somatic variant call files (VCFs) were filtered with read-depth greater than or equal to 50× to account for tumor heterogeneity. Somatic variants were called using the snpEFF-v4.3t tool^42^ and analyzed downstream using OpenCravat-v2.2.225^43^. Rare variants that have less than 1% MAF in gnomAD were retained, and the rest were filtered out. SNV and tumor mutational burden were visualized using Maftools package^44^ using R 4.1.0.

### Mutational signature profile

MutationalPatterns 3.11.0 package was used to identify NMF and comparing the profile with predefined SIGNAL and COSMIC mutation signatures. Default parameters were used in the analysis (https://bioconductor.org/packages/release/bioc/html/MutationalPatterns.html). SNVs called by at least two of three variant calling algorithms were filtered by a read-depth of 50× or higher. This analysis included VAF > 5% and rare variants with < 1% MAF (Supplementary Table 1).

### Supplementary Information

Supplementary Figures.

Supplementary Tables.

## Data Availability

The lists of variants and classifications are available in Supplementary Table 2. The list of germline mutation reference data can be accessed via the gnomAD browser (https://gnomad.broadinstitute.org/downloads). The EAC sequencing datasets generated and analyzed during the current study are available in the BioProject database under accession PRJNA1077968. TCGA Esophageal cancer carcinoma cohort data can be accessed via the National Cancer Institute GDC Data portal (https://portal.gdc.cancer.gov/projects/TCGA-ESCA).

## Code availability

Whole-exome sequences were aligned, variants were called using a GATK-based pipeline (https://gatk.broadinstitute.org/hc/en-us). FastQC package was used to assess the alignment quality (http://www.bioinformatics.babraham.ac.uk/projects/fastqc). GATK Funcotator tool was used for variant annotation and quality control. Somatic variants were called using the snpEFF-v4.3t package (https://pcingola.github.io/SnpEff/), and analyzed downstream using OpenCravat-v2.2.225 package (https://open-cravat.readthedocs.io/en/latest/quickstart.html). Maftools R package was used to summarize and visualize MAF files using R 4.1.0. (https://github.com/PoisonAlien/maftools). MutationalPatterns 3.11.0 package was used to identify SIGNAL and COSMIC mutational signatures and identity mutational signatures based on nonnegative matrix factorization (NMF). (https://bioconductor.org/packages/release/bioc/html/MutationalPatterns.html). MutSigCV tool (https://github.com/getzlab/MutSig2CV/blob/master/README.md) was used for identifying EAC driven genes.
