# Supplementary material for: Somatic mutations of esophageal adenocarcinoma: a comparison between Black and White patients
Source: Sci Rep. 2024 Apr 18;14:8988. doi: 10.1038/s41598-024-59257-3 (PMC11026501; doi:10.1038/s41598-024-59257-3)
Supplement: Supplementary file 1 — Supplementary Figures. [file 41598_2024_59257_MOESM1_ESM.docx]

**Supplementary Figure legend**

**Supplementary Figure 1**. Mutational Signatures based on COSMIC Signatures. (a) Dotplot of the relative contribution of COMIC signatures. The color of the dot shows the percentage of iterations in which the signature is found (contribution > 0), and the size of the dot represents the average contribution of that signature (in the iterations in which the contribution was higher than 0).

**Supplementary Figure 2.** Mutational Signatures were identified using a non-negative matrix factorization (NMF) algorithm after excluding TMB outlier samples (B6 and B9). (a) The relative contribution of each indicated trinucleotide change to the two mutational signatures that were identified by NMF analysis of the somatic mutation of the EAC samples (b) Bar graphs show the percentage of the two signatures in the EAC tumor samples.

**Supplementary Figure 3**. Single nucleotide variants and InDels of EAC. Oncoplot by maftools visualized mutations of missense, frameshift InDels, nonsense, and splice site. The six genes according to the p-value < 0.05 from Fisher’s exact test for comparing the number of patients affected by individual genes between Black and White patients.

**Supplementary Figure 1**. Mutational Signatures based on COSMIC Signatures. (a) Dotplot of the relative contribution of COMIC signatures. The color of the dot shows the percentage of iterations in which the signature is found (contribution > 0), and the size of the dot represents the average contribution of that signature (in the iterations in which the contribution was higher than 0).

**a.**


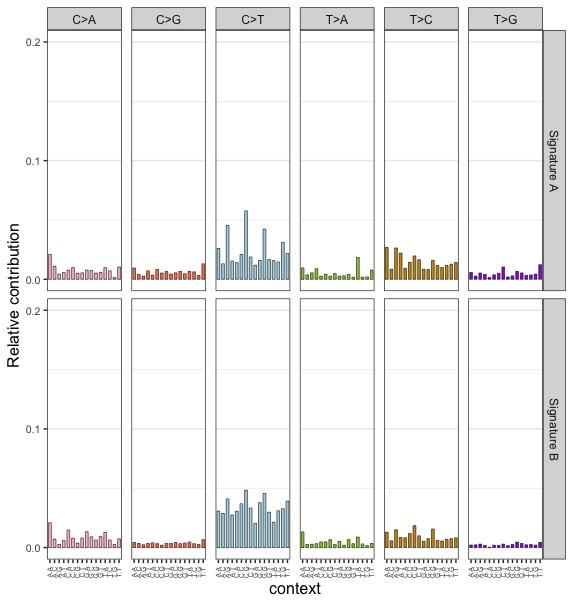
**b.**

**c.**

**Supplementary Figure 2.** Summary of exome sequencing and tumor mutation burden (TMB) after excluding TMB/Mb outlier samples (B6 and B9). (a) Number and types of variants found in each sample. The red dashed line presents the median of the total number of variants (Black=1,047 White=735.5). (b) Mutational Signatures were identified using a non-negative matrix factorization (NMF) algorithm. The relative contribution of each indicated trinucleotide change to the two mutational signatures that were identified by NMF analysis of the somatic mutation of the EAC samples (c) Bar graphs show the percentage of the two signatures in the EAC tumor samples.

**Supplementary Figure 3**. Single nucleotide variants and InDels of EAC. Oncoplot by maftools visualized mutations of missense, frameshift InDels, nonsense, and splice site. The six genes according to the p-value < 0.05 from Fisher’s exact test for comparing the number of patients affected by individual genes between Black and White patients.
